# Editorial: Cortico-hippocampal interactions during learning and memory

**DOI:** 10.3389/fnbeh.2024.1454782

**Published:** 2024-08-13

**Authors:** Travis P. Todd, Christian Bravo-Rivera, Karyn M. Frick, Sharon C. Furtak

**Affiliations:** ^1^Department of Psychological Science, University of Vermont, Burlington, VT, United States; ^2^Departments of Psychiatry and Anatomy & Neurobiology, University of Puerto Rico School of Medicine, San Juan, Puerto Rico; ^3^Department of Psychology, University of Wisconsin-Milwaukee, Milwaukee, WI, United States; ^4^Department of Psychology, California State University Sacramento, Sacramento, CA, United States

**Keywords:** hippocampus, cortex, learning, memory, conditioning

## Introduction

Although it has long been recognized that interactions among the hippocampus, the parahippocampal region, and the neocortex support learning and memory (see Eichenbaum, 2000), the cellular and circuit mechanisms through which they interact remain unclear. Indeed, ongoing research seeks to understand the unique contributions that these distinct areas make to learning and memory, as well as how interactions at the circuit and network levels support these cognitive functions. The main goal of this Research Topic is to shed new light regarding how cortico-hippocampal interactions give rise to learning and memory. Such research includes direct manipulation of cortico-hippocampal circuits in learning and memory, or indirect tests of these circuits via local manipulation of a targeted brain region. To that end, this Research Topic includes eight papers, that can be broadly classified into three collections that: (1) provide new perspectives on the hippocampus, (2) elucidate cortical contributions to aversive memory, and (3) present theoretical and conceptual reviews of cortico-hippocampal interactions. Collectively these papers highlight the importance of investigating how brain structures work in collaboration to support learning and memory, which represents a more powerful approach than examining each region in isolations. This more global perspective facilitates the ability to examine dynamic functions throughout cognitive brain circuits as task demands change over time.

## New perspectives on the hippocampus

The first set of papers explored the role of the hippocampus and related brain regions in memory related behavior, providing new insights into roles for ventral hippocampal connections to the prelimbic cortex and amygdala, as well as the role of hippocampal neurogenesis. The first paper, which examined the vHipp in male rats, focused on the involvement of the vHipp-prelimbic cortex (PL) pathway in context-specific operant responding (Thomas et al.). Through chemogenetic inhibition, the researchers selectively inhibited either the vHipp or its direct projections to the PL during ongoing behavior. Results showed that only vHipp inhibition, and not its projections to the PL, reduced context-specific operant responding, indicating that the vHipp plays a crucial role in context-mediated behavioral responses independent of its direct projections to the PL.

In the second paper, Ritger et al. investigated how elevated fear states influence the engagement between the ventral hippocampus (vHipp) and the basolateral amygdala (BLA) in male rats. Using *in vivo* extracellular electrophysiology, the researchers found increased spontaneous firing in the BLA and a heightened responsiveness to vHipp stimulation during elevated fear states. These results suggest that the enhanced activity of the vHipp-BLA pathway supports the reconsolidation and retrieval of elevated fear memories, underscoring the importance of the vHipp-BLA pathway in modulating fear responses and providing insight into how fear memories are processed and maintained in the brain. Together with the results of Thomas et al., these studies provide a comprehensive understanding of how the ventral hippocampus and its connections to other brain regions, such as the BLA and PL, orchestrate complex fear and context-related processes, offering valuable insights into the neural mechanisms underlying fear modulation and cognitive adaptability.

The final paper in this Research Topic explored the role of hippocampal neurogenesis in cognitive flexibility in male rats, particularly in distinguishing between aversive and safe contexts (Martínez-Canabal et al.). The investigators found that environmental enrichment and the administration of memantine, which enhances neurogenesis, improved the rats' ability to perform fear discrimination tasks. Conversely, reducing neurogenesis with methotrexate eliminated this enhanced cognitive flexibility, highlighting the critical role of new neuron formation in the hippocampus for adaptive fear responses. Collectively, these studies highlight the pivotal role of hippocampal circuitry in modulating fear-related behaviors and cognitive flexibility.

## Cortical contributions to aversive memory

The second collection of papers focused on cortical contributions to aversive memory and highlighted the role of the retrosplenial cortex (RSC), a region of association cortex that interacts with the hippocampus during normal memory processing (see Miller et al., 2014). Two manuscripts in this Research Topic examined the contribution of the RSC to inhibitory avoidance learning and memory. In the first paper, Hoxha et al. examined the expression of the immediate early gene zif268 and perineuronal nets (PNNs) in the RSC in male and female rats. In one experiment, zif268 was elevated following training in both the anterior and posterior RSC, indicating the RSC is engaged during memory formation. PNNs were reduced in the posterior RSC, which may be indicative of memory consolidation. Next, Hoxha et al. examined the role of the RSC in generalization and discrimination of inhibitory avoidance. To do so, rats were trained in one context (either a light or dark chamber), and then tested in either the same or a different context. Hoxha et al. observed that rats showed robust *discrimination* when trained in the dark context and tested in the light context. However, *generalization* was observed when animals were trained in the light but tested in the dark context. Following these tests, zif268 and PNNs expression were assessed, including an analysis in which subjects were classified according to their behavioral performance. When considering the overall pattern of data, Hoxha et al. concluded that anterior RSC activity tends to correspond with learned avoidance, but posterior activity seems to correspond with generalization of avoidance.

In the second paper examining inhibitory avoidance, Pastor and Katche examined the contribution of cholinergic signaling in the RSC to different phases of processing in male rats. In these experiments, an α7 receptor antagonist (methyllycaconitine: MLA) was infused into the anterior portion of the RSC, either prior to acquisition (learning), just after acquisition (consolidation), or prior to testing (retrieval). Interestingly, these authors reported that MLA infusion into the anterior RSC impacted behavior only if infusions occurred prior to acquisition or prior to testing, suggesting a role for nicotinic acetylcholine receptors in the RSC during memory acquisition and retrieval.

Finally, Cheng et al. reviewed the contribution of the RSC in aversive conditioning paradigms, focusing on discriminative avoidance learning, and Pavlovian fear conditioning. Whereas, the RSC has often been most associated with contributions to contextual learning as well as spatial navigation and cognition, Cheng et al. described many instances in which the RSC contributes to learning and memory for discrete auditory and visual cues that predict aversive outcomes. Much of the literature reviewed was published by Gabriel and colleagues (for a review see Gabriel, 1993), who in an extensive body of research examined neural activity in the RSC, as well as the impact of RSC lesions, during wheel-turn avoidance in rabbits. To integrate the avoidance and Pavlovian literature, Cheng et al. finished by speculating about the types of information encoded by the RSC in both paradigms, the role of the RSC in memory storage, and how RSC contributions to learning and memory for discrete cues that predict aversive outcome might be tied to its known role in contextual learning and memory.

## Theoretical and conceptual review

Finally, in the last group of papers, the authors address theoretical and conceptual aspects of cortico-hippocampal contributions to learning and memory. In a paper by Krasne and Fanselow, a new theoretical model of remote memory is proposed (BaconREM), which is an extension of a previously published Bayesian model of context fear conditioning (Krasne et al., 2015). This model accounts for several phenomena related to contextual fear generalization. Importantly, this theoretical model of remote memory lends novel insight into neocortical-hippocampal interactions that predict when and how the hippocampus supports encoding and updating contextual aspects of remote memories by working collaboratively with neocortical regions (Krasne and Fanselow). Theoretical models continue to be an essential component of understanding behavioral phenomenon and creating experimentally testable predictions of system level dynamics. Furthering our conceptual understanding of cortico-hippocampal interactions in learning and memory, a paper by Plas et al. reviews adaptive regulation of fear extinction memories supported by prefrontal-hippocampal interactions. The review highlights both historical and recent research on the neural mechanism underlying fear extinction memories. For example, the authors draw attention to recent optogenetic research distinguishing subregions of the dentate gyrus in contextual fear memories and examine in great depth recent research demonstrating the importance of the nucleus reuniens of the thalamus, a region that interconnects the hippocampus and the medial prefrontal cortex, in fear extinction memories (Plas et al.). The review also examines the modulatory effects of stress on the acquisition and retrieval of fear extinction memories via activation of the locus coeruleus. The conceptual review expands traditional ideas of how the hippocampus engages with both cortical and subcortical regions to support learning and memory.

## Concluding remarks

Advances in circuit- and network- level analysis techniques have pushed the scientific community to consider the function of the hippocampus in a more holistic manner. The field has moved beyond focus on a single structure to hypotheses that reflect the function of a structure within a network of regions with a common purpose. This global perspective has produced computation and experimental research that, while preserving the importance of the hippocampus in learning and memory, suggests a dynamic functional role as the hippocampus works collaboratively with cortical and subcortical structures to support various types of learning and memory. The collection of papers in the current Research Topic reflects not only an evolution of the field toward a more global role for the hippocampus in brain-wide memory circuits, but also the cutting-edge technical advancements that have made this broader perspective possible.

## Author contributions

TT: Writing – original draft, Writing – review & editing. CB-R: Writing – original draft, Writing – review & editing. KF: Writing – original draft, Writing – review & editing. SF: Writing – original draft, Writing – review & editing.
